# An Experimental Platform for Tomographic Reconstruction of Tissue Images in Brightfield Microscopy

**DOI:** 10.3390/s23239344

**Published:** 2023-11-23

**Authors:** Panteleimon Koudounas, Efthymios Koniaris, Ioannis Manolis, Panteleimon Asvestas, Spiros Kostopoulos, Dionisis Cavouras, Dimitris Glotsos

**Affiliations:** 1 Medical Image and Signal Processing Laboratory, Department of Biomedical Engineering, University of West Attica, Egaleo, 12243 Athens, Greece; pkoudounas@uniwa.gr (P.K.); pasv@uniwa.gr (P.A.); skostopoulos@uniwa.gr (S.K.);; 2 Department of Pathology, Hippocration General Hospital, 11527 Athens, Greece; ethimiok@hotmail.com (E.K.); imanolis@uniwa.gr (I.M.)

**Keywords:** cancer, histopathology, optical microscopy, 3D tissue volume imaging

## Abstract

(1) Background: Reviewing biological material under the microscope is a demanding and time-consuming process, prone to diagnostic pitfalls. In this study, a methodology for tomographic imaging of tissue sections is presented, relying on the idea that each tissue sample has a finite thickness and, therefore, it is possible to create images at different levels within the sample, revealing details that would probably not be seen otherwise. (2) Methods: Optical slicing was possible by developing a custom-made microscopy stage controlled by an ARDUINO. The custom-made stage, besides the normal sample movements that it should provide along the x-, y-, and z- axes, may additionally rotate the sample around the horizontal axis of the microscope slide. This rotation allows the conversion of the optical microscope into a CT geometry, enabling optical slicing of the sample using projection-based tomographic reconstruction algorithms. (3) Results: The resulting images were of satisfactory quality, but they exhibited some artifacts, which are particularly evident in the axial plane images. (4) Conclusions: Using classical tomographic reconstruction algorithms at limited angles, it is possible to investigate the sample at any desired optical plane, revealing information that would be difficult to identify when focusing only on the conventional 2D images.

## 1. Introduction

Diagnostic pathology [1] usually involves the diagnosis of various diseases on the basis of tissue, cellular, or other kinds of biological material under the optical microscope. Although modern non-interventional medical imaging technologies may provide indications regarding the presence of a disease, most of the time, for serious diseases such as cancer, confirmation is still based on findings at the microscopic level [2]. 

Microscopic examination is not an easy task [1]. Its accuracy depends on many factors, such as accurate sampling of biological material, appropriate preparation of the sampled material for microscopy viewing, and accurate visual evaluation by the expert physician. All of these factors (sampling, preparation, interpretation) have been proven to invoke diagnostic errors, which may affect outcomes in patient management [3,4,5,6]. Especially in cancer, diagnostic errors may determine whether a patient will survive or not [7]. 

In order to safeguard the correctness and validity in microscopy examination, several methods have been proposed in the literature, target different aspects of the process. First, sampling of biological material should originate from the most representative region of the pathology. This may be secured, for example, by image-guided biopsy [8,9] using ultrasound or other technologies. Second, the preparation of the material should ensure that the most informative features of the pathology will be visually identifiable when viewed under the microscope. For example, in tissue biopsies, several chemicals are utilized to paint specific parts of the biological material (e.g., chromatin, proteins, etc.) with a distinct color [1]. Third, the expert physician should be able to identify and interpret the most important findings on the magnified sample. This process is not straightforward; rather, it is time consuming and cumbersome. Especially for tissue biopsies that involve multiple sections on multiple slides, thus requiring the reviewing of hundreds/thousands of images at different magnifications, diagnostic pitfalls might arise due to the following two reasons: a/the expert physician might miss the most representative region of the sample, and b/the expert physician might not interpret correctly the imaging findings due lack of experience or skills [10,11,12,13,14,15]. To secure accurate results, a/automated systems have been proposed, able to scan the full extent of the biological material and guide the expert physician to the most representative regions of the pathology [16,17], and b/provide second opinion diagnostic interpretations based on automated image analysis (decision support systems) [18,19,20].

Diagnostic pathology relies completely on 2D images. The physical material is placed on the microscopy slide, upon which observation is performed on a 2D plane. Especially for tissue biopsies, even though the original tissue material is 3D, before observing the sample under the microscope, the material is prepared/cut into 2D slices with the microtome, and after appropriate chemical processing (i.e., staining), it is placed on the microscopy slide, upon which observation is, again, performed on a 2D plane. 

However, 2D images are an approximation of 3D reality and may lead to less accurate conclusions. This issue has long been investigated in the field of medical imaging at the ‘macroscopic’ scale, i.e., the scale of the human body. Consider, for example, the X-ray computed tomography (CT) systems, which were developed to produce consecutive 2D optical slices of the patient’s body. These slices, when stacked together, produce a 3D representation of the patient’s volume, which has proven a more accurate approach than the standard 2D projection X-ray radiography [21]. Would it be useful to expand this idea to diagnostic pathology and, in particular, to the microscopic examination of tissue material, which, by definition, is a 3D material? We have provided a preliminary answer, a proof of concept, in our previous study, that such a quest is meaningful [22]. Our approach involved the physical slicing of the tissue biopsy material into consecutive 2D slices, production of digital 2D images of each physical 2D slice, and reconstruction of the 3D volume by placing consecutive digital 2D images one next to the other. To investigate whether the generated 3D volumes might have any clinical value, evaluation from expert histopathologists was sought. According to the experts’ opinions, the 3D volumes provided additional diagnostic information that was not identifiable in the 2D images, verifying the proof of concept, i.e., that it would be meaningful to produce 3D volume images in diagnostic pathology since it may reveal new information, difficult to spot in the 2D domain. The problem with the above-mentioned approach is that the sample needs to be physically sliced, processed, and stained, increasing the overall time, cost, and complexity of the process. 

In this work, we extend our efforts towards 3D tissue volume imaging in microscopy using optical slicing of the sample, instead of the physical slicing approach that we followed in our previous study [22]. Optical slicing was possible by developing a custom-made microscopy stage that, besides the normal sample movements that it should provide along the x-, y-, and z- axes, it may also rotate the sample around the horizontal axis. This rotation enables the conversion of the optical microscope into a CT geometry, enabling optical slicing of the sample using projection-based tomographic reconstruction algorithms. To the best of the authors’ knowledge, this is the first time such a method is presented in the literature. 

## 2. Materials and Methods

The materials comprised tissue samples from the archival material at the Pathology Department of the General Hospital of Athens “Hippokratio”. The process of data collection and the overall research protocol received approval from both the Scientific Council of the General Hospital of Athens “Hippokratio” (Approval Number 65/8-11-2021) and the Research Ethics Committee of the University of West Attica (Approval Number 108080/30-11-2021).

Each tissue sample underwent sectioning with a microtome to produce sections that were 10 μm thick. Subsequently, these tissue sections were stained using hematoxylin and eosin (H&E), numbered, and affixed to microscope slides. For capturing images, a digital light microscopy imaging system (LEICA DM 2500) equipped with a LEICA DFC 420 C color camera was employed. The illumination source utilized was a 12 V, 10 W tungsten-halogen lamp. Images were digitized with a resolution of 1728 × 1296 pixels (with a pixel size of 2.78 μm × 2.78 μm) and a color depth of 24 bits. Lenses with a cumulative magnification factor of ×100 were employed (objective lens ×10 and zoom lens similar to eyepieces ×10). The objective lens had a numerical aperture equal to 0.25 and depth of focus equal to 8.5 μm. 

According to the proposed methodology, images of a tissue section are collected at different angles. These images play the role of projections and are used to generate images at different tissue depths and in different planes (axial plane, coronal plane, and oblique plane). The methodology involves the following steps:

- Acquisition of images at different angles using a customized experimental microscope stage adjusted to a commercial microscope
- Alignment of the images
- Tomographic reconstruction

### 2.1. Image Acquisition Setup

**Figure 1:**
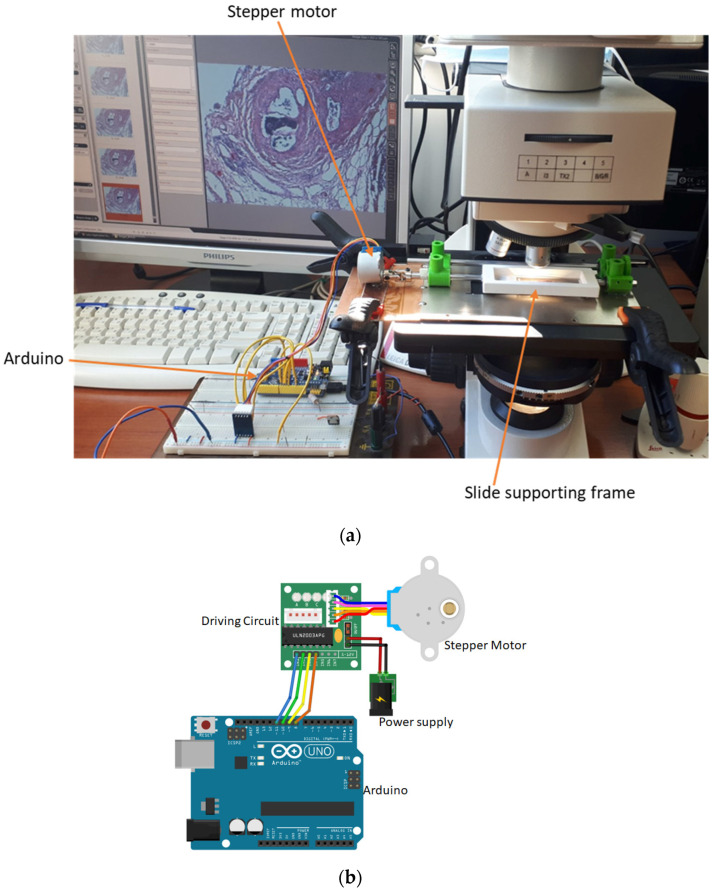
(**a**) Setup for the acquisition of cross-sectional images of tissue at various angles. (**b**) Schematic diagram of the electronics of the experimental setup.

The setup used to capture the images comprised a custom-developed experimental microscope stage adjusted to a commercial microscope (LEICA DM 2500) (Figure 1a). The experimental stage was designed to allow for rotation around the horizontal axis of the microscope slide and movement along the x, y, and z axes by a stepper motor controlled by Arduino (Figure 1b). 

**Figure 2:**
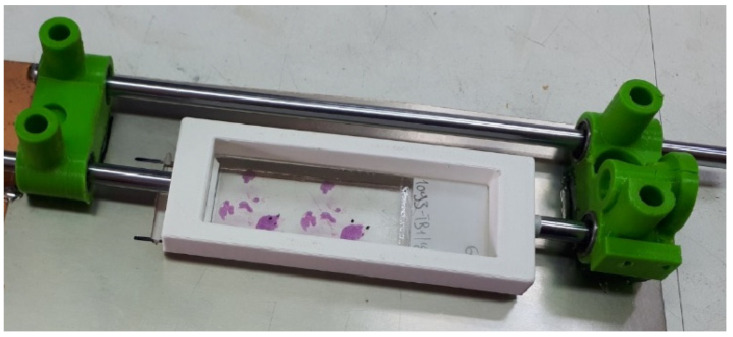
Slide supporting frame with tissue sections.

The microscope slide is placed at a specially designed frame (Figure 2).

The Arduino and the microscope camera are connected to a computer, which runs a program written in Python. This program instructs the Arduino to rotate the slide with given angular steps. At each different angular position, an image of the sample is collected. This custom-made operation simulates the ‘step and shoot’ operation of an X-ray Computed Tomography scanner (with the difference being that the source (illumination lamp) and the detector (camera) remain fixed while the sample is rotated).

**Figure 3:**
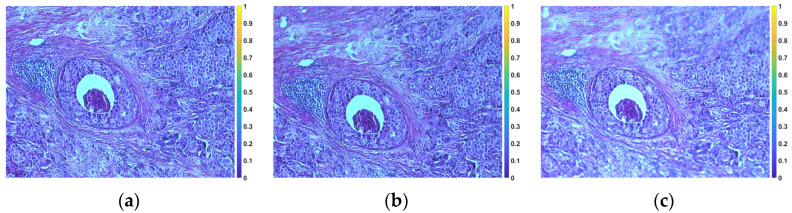
Microscope images of the rotation of a tile. (**a**) Without rotation. (**b**) 5° rotation. (**c**) 10° rotation.

Figure 3 shows representative images of tissue without rotation (horizontal position) and with 5° and 10° tile rotation, respectively.

### 2.2. Image Registration

Ideally, the rotation of the slide containing the tissue should be around the horizontal axis passing through the center of the tile, otherwise the tissue will appear displaced in the corresponding image. As there may be small deviations from the ideal axis of rotation in the proposed setup, each image is aligned with the image corresponding to the horizontal position (0°) (reference image) using the phase correlation method [23]. Aligning images by phase correlation is a technique particularly useful when it comes to images that have undergone translation, rotation or scaling transformations. Phase correlation works by analyzing the frequency domain representation of images to determine their spatial displacement. The steps involved are as follows:

- Transforming images in the frequency domain: images are transformed from the spatial domain to the frequency domain using the fast Fourier transform (FFT).
- Calculation of the cross-power spectrum: the cross-power spectrum of the two transformed images is calculated by multiplying the Fourier transform coefficients of one image by the complex conjugates of the Fourier transform coefficients of the other image. The result represents the similarity between the two images in the frequency domain.
- Calculation of the phase correlation: The phase correlation is obtained from the cross-power spectrum by taking the inverse Fourier transform. The phase correlation image encodes information about the relative displacement between the two input images. The peak of the phase correlation image indicates the offset between the images.
- Finding the peak: The peak of the phase correlation image, which corresponds to the relative offset between the two images, is located. The location of this vertex provides the horizontal and vertical offset required to align the images.

**Figure 4:**
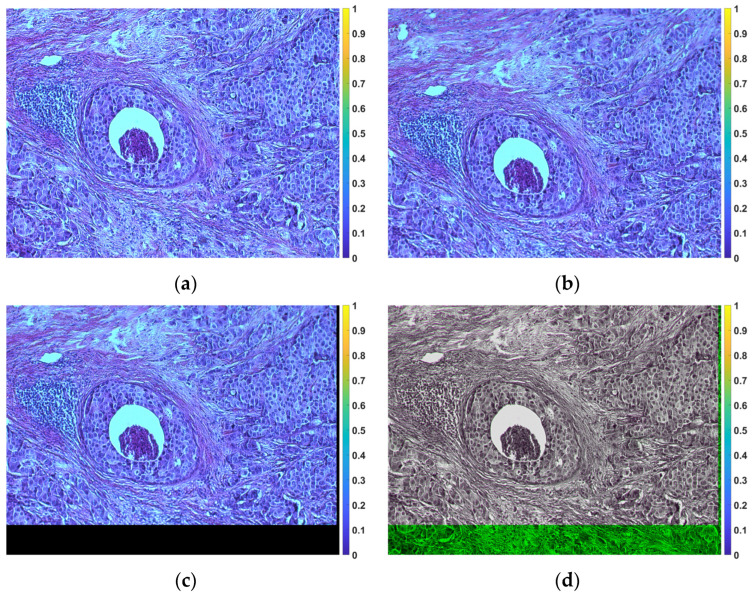
Example of registration. (**a**) Reference image (the tile in the horizontal position). (**b**) Image for 5° rotation before registration. (**c**) Image after registration. (**d**) Fusion of the reference image with the registered image.

Figure 4 shows an illustrative example of alignment.

### 2.3. Image Reconstruction

From the previous step, a set of 2D aligned grayscale images from the original 3D sample is obtained. If *c_j* (*j* = 1, 2, ...) denotes any given column of each image, then the values of column *c_j* can be considered as (optical) projections of the original 3D sample at a given angular position. With the assistance of these optical projections, it becomes feasible to produce 2D cross-sectional images of the sample (optical slices) along the z-axis using established reconstruction techniques commonly employed in analogous problems such as in X-ray CT. Some such methods are the filtered backprojection (FBP) or algebraic reconstruction techniques (ART) [24].

FBP is a fundamental technique in medical imaging, particularly in X-ray CT and other imaging modalities. It is used to reconstruct cross-sectional images of an object, such as a human body, from a series of 2D X-ray projections or other types of projection data. The acquired projection data is subjected to the Radon transform, transforming the 2D projection data into a 1D representation. The Radon-transformed data is then filtered using a specific mathematical filter, such as a ramp or Shepp-Logan filter. This filtering step is crucial to improve image quality by reducing artifacts and enhancing the visibility of structures in the reconstructed image. After filtering, the filtered data is backprojected. This involves taking the filtered 1D data and mathematically “projecting” it back into 2D space. This process is repeated for multiple angles, and the results are summed together to build up the final 2D image. The summation of the backprojected images from different angles yields a 2D image that represents a cross-section of the object.

The ART is another method for reconstructing images from projection data, similar to the FBP technique. The key characteristic of the ART is its iterative nature. It does not rely on a single filtering and backprojection step as does FBP; instead, it iteratively updates the image estimate to bring it closer to the true object. The basic steps in each iteration are as follows:

- Forward Projection: The current image estimate is projected onto the data space to generate a set of synthetic projections.
- Residual Calculation: The difference between the measured projections and the synthetic projections is calculated. This represents the error in the current image estimate.
- Backprojection Correction: The error is backprojected into the image space and scaled by a relaxation parameter.
- Image Update: The error-corrected backprojection result is added to the current image estimate to obtain an updated image estimate.
- The iterative process continues for a predefined number of iterations or until a convergence criterion is met, indicating that the image estimate is sufficiently close to the true object. The ART is particularly useful when dealing with limited-angle or sparse data, as it can gradually improve the image estimate by iteratively fitting the available data. It is more computationally intensive than FBP but can produce better image quality in certain situations.

## 3. Results

**Figure 5:**
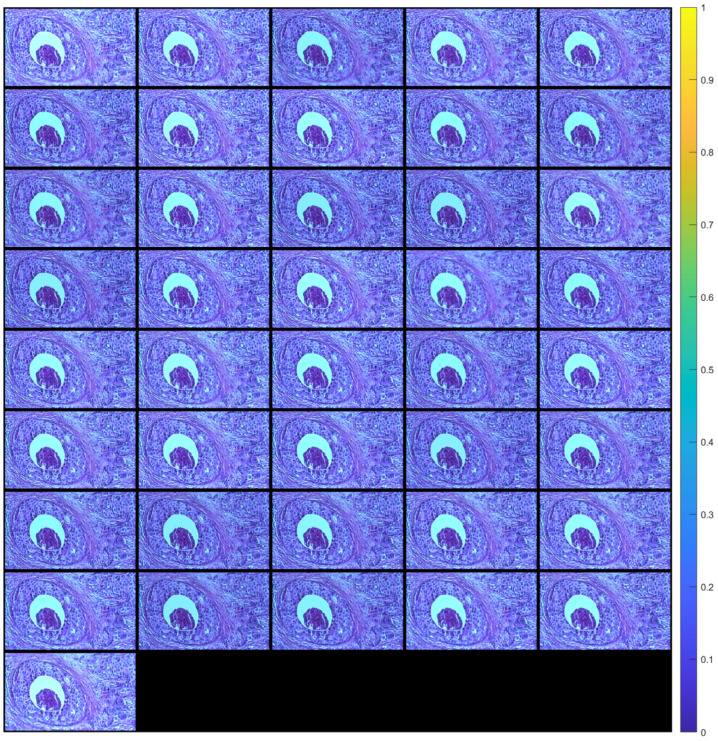
Images (after registration) of tissue section for angles from −10° to +10° with a 0.5° step. The images are arranged in ascending rotation angle row-wise.

Figure 5 shows the images of a tissue section (after alignment) for a range of angles from −10° to +10° with a 0.5° step (41 images in total). Each aligned image was cropped to remove the black peripheral region resulting from applying the transformation (see Figure 4c). The cropping was done in such a way that all images eventually have the same dimensions.

**Figure 6:**
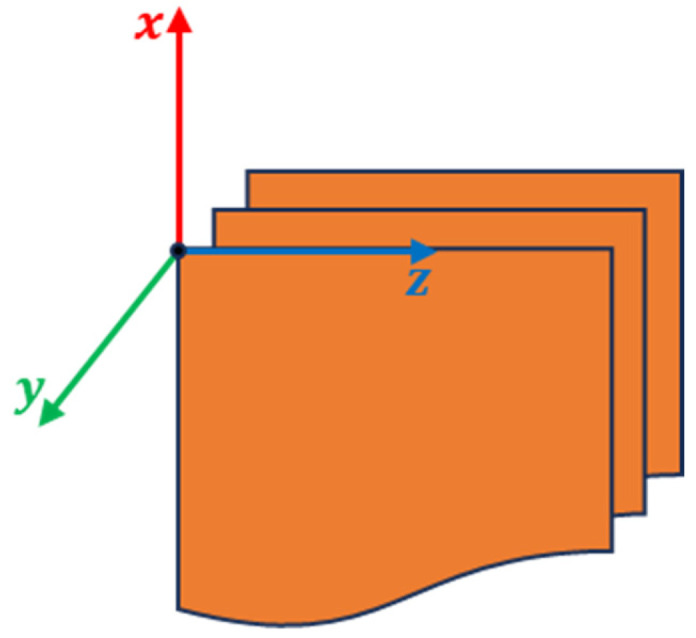
Definition of coordinate system.

A 3D cartesian axis system was defined, where the z-axis is in the direction of the rows of the images, the x-axis is in the direction of the columns of the images, and the y-axis is perpendicular to the plane of the images (Figure 6). 

Then, for each column of the images (i.e., for a fixed z), reconstruction was performed using two methods:

- FBP
- ART

In this way, images in the x-y plane (axial plane) were obtained. The number of images were as many as the number of columns of the original images. By rearranging the rows and columns of these images, images in the y-z (sagittal) and x-z (coronal) planes were obtained.

**Figure 7:**
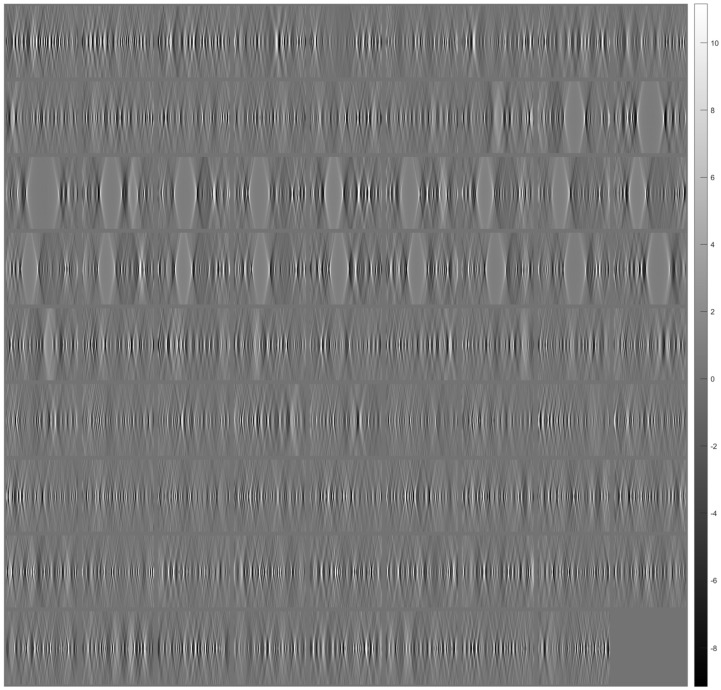
Tomographic imaging in the coronal plane using the FBP technique.

**Figure 8:**
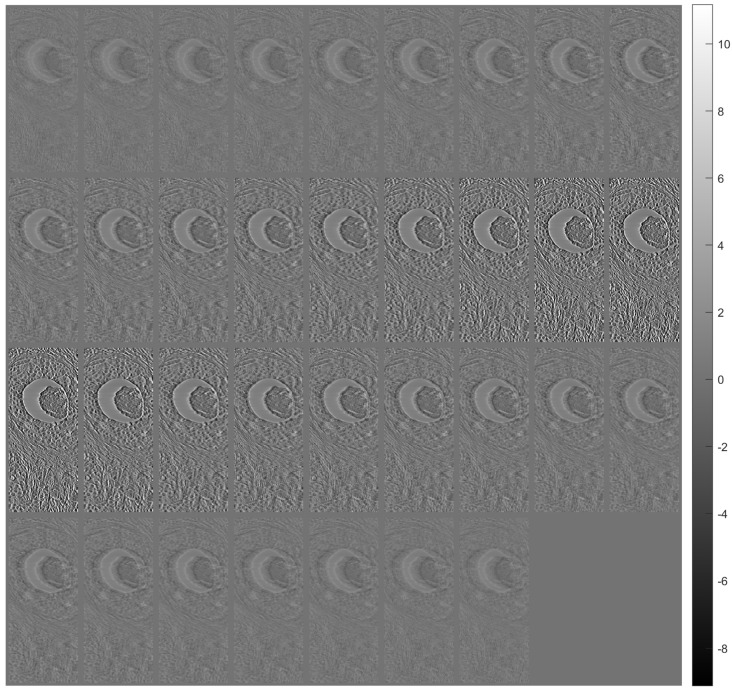
Tomographic imaging in the axial plane using the FBP technique.

**Figure 9:**
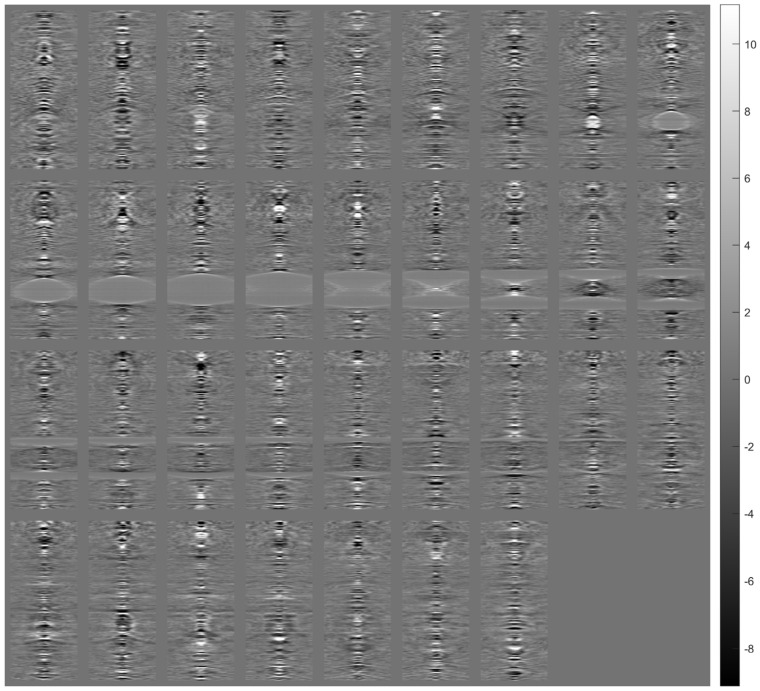
Tomographic imaging in the sagittal plane using the FBP technique.

Figure 7 shows tomographic imaging in the coronal plane using the FBP technique. Figure 8 and Figure 9 show tomographic imaging in the axial and sagittal planes, respectively. Note that the images have been reduced in size to save space.

**Figure 10:**
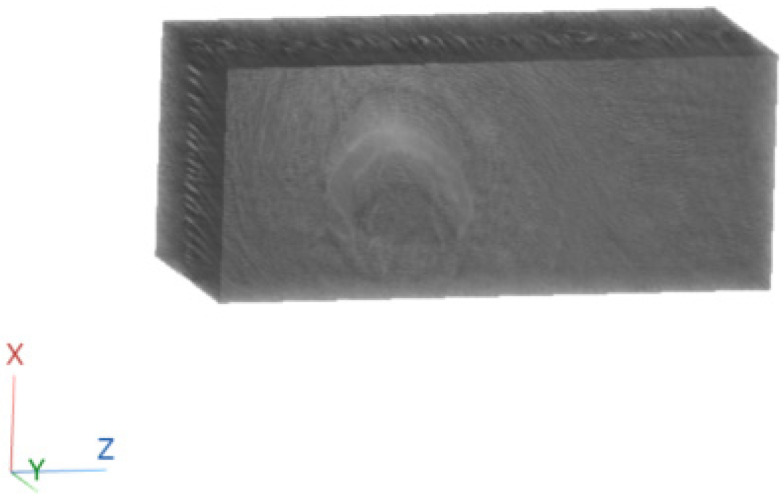
Three-dimensional reconstruction of a tissue section using FBP.

To better illustrate the image content of Figure 7, Figure 8 and Figure 9, all the axial slices produced by the proposed methodology were used to generate Figure 10, which shows the 3D representation of the tissue section by the FBP technique. 

**Figure 11:**
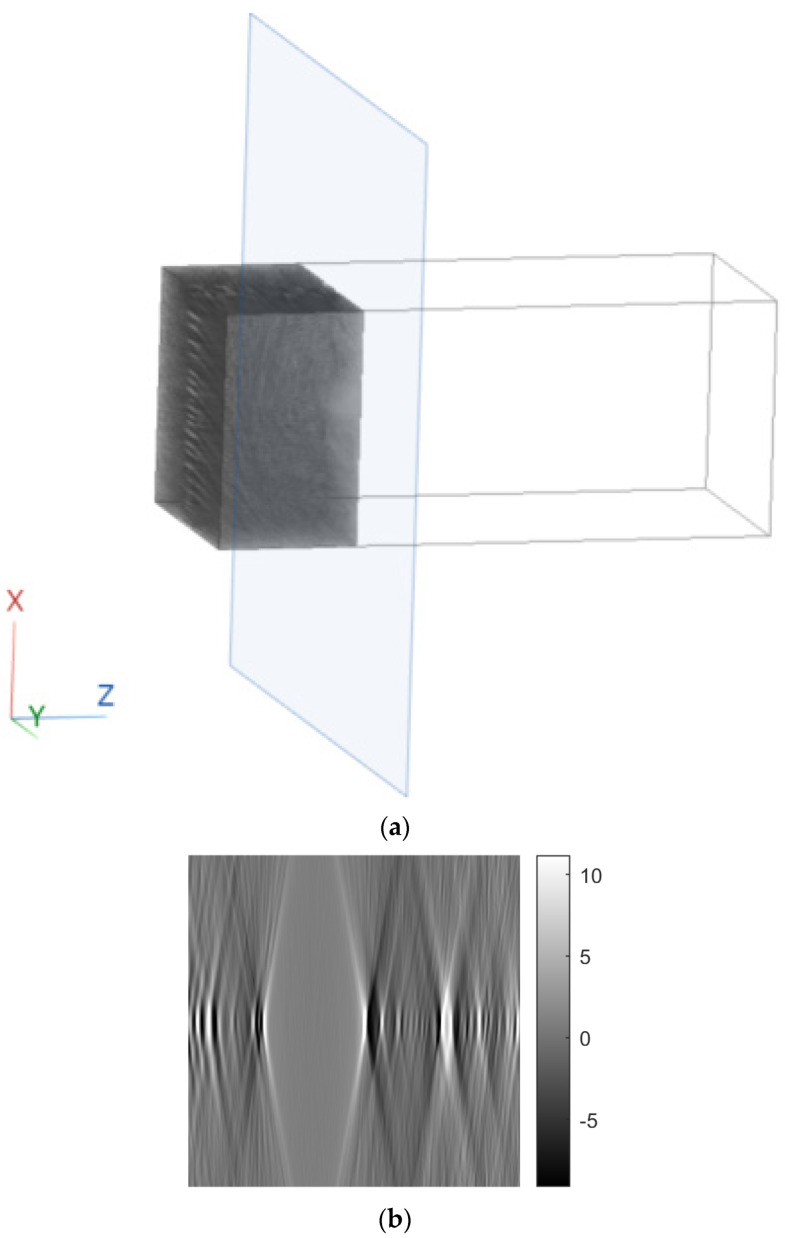
Example of an image in the axial plane using FBP. (**a**) The plane in which the image was taken. (**b**) The image.

**Figure 12:**
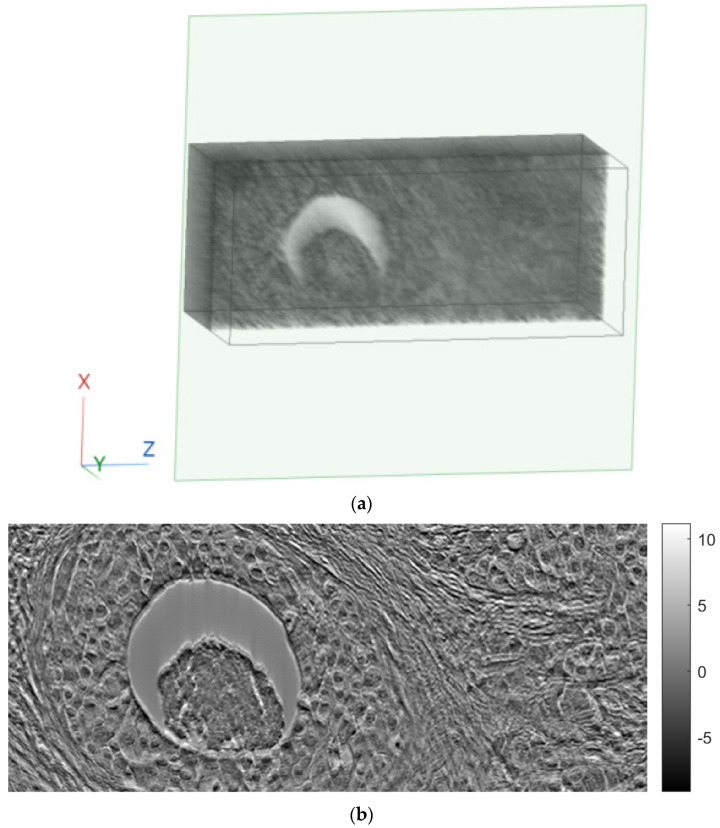
Example of an image in the coronal plane using FBP. (**a**) The plane in which the image was taken. (**b**) The image.

**Figure 13:**
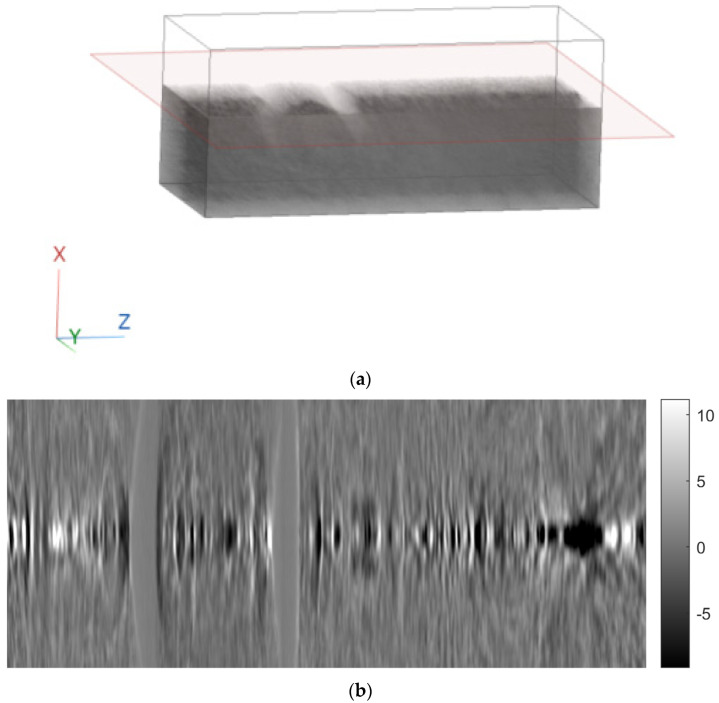
Example of an image in the sagittal plane using FBP. (**a**) The plane in which the image was taken. (**b**) The image.

Figure 11, Figure 12 and Figure 13 show examples of images generated in the axial, coronal, and sagittal planes, respectively. The 2D images in Figure 11b, Figure 12b and Figure 13b were obtained by slicing the volume at each particular plane, as illustrated at Figure 11a, Figure 12a and Figure 13a, respectively.

**Figure 14:**
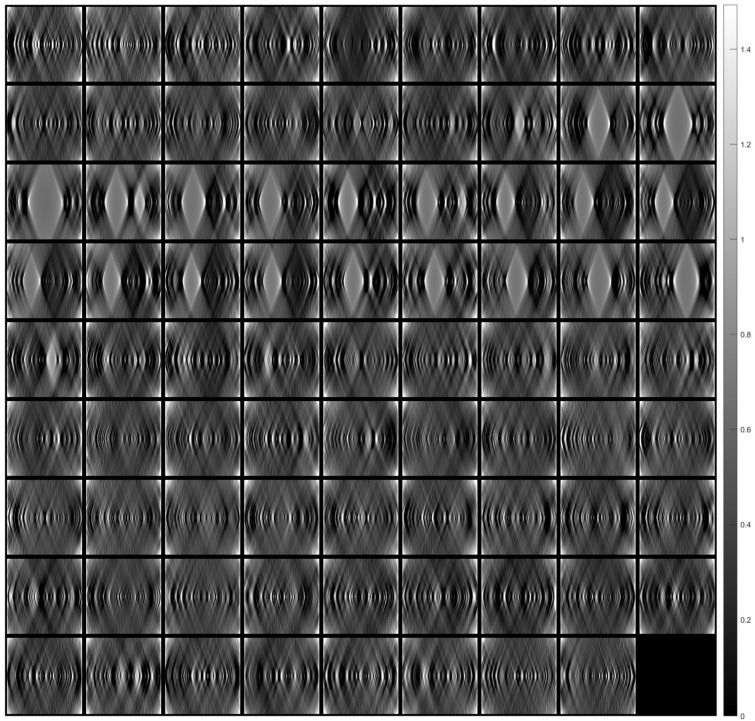
Tomographic imaging in the axial plane using ART.

**Figure 15:**
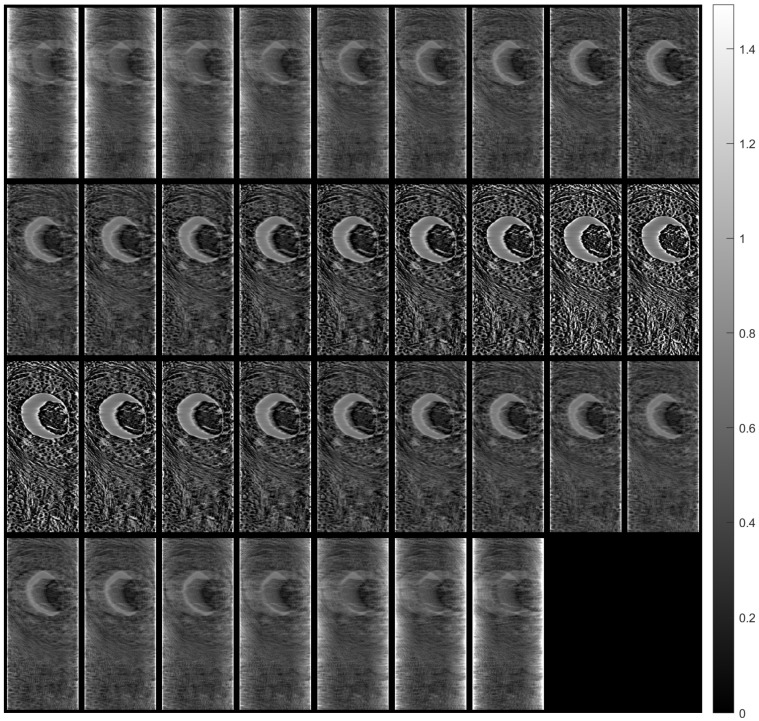
Tomographic imaging in the coronal plane using ART.

**Figure 16:**
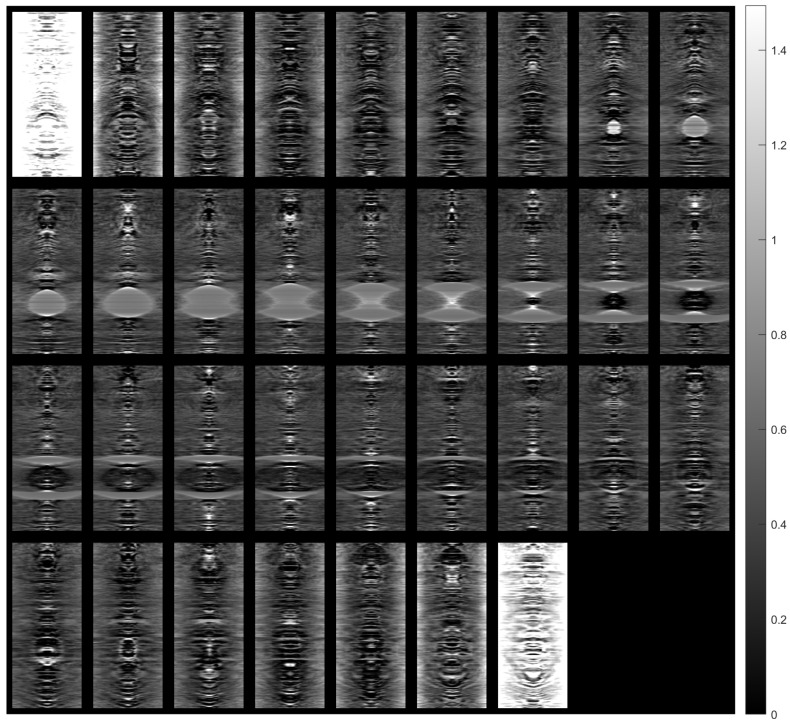
Tomographic imaging in the sagittal plane using ART.

Figure 14 shows images of tomographic imaging in the axial plane using ART, using 10 iterations. Figure 15 and Figure 16 show images in the coronal and sagittal planes, respectively.

**Figure 17:**
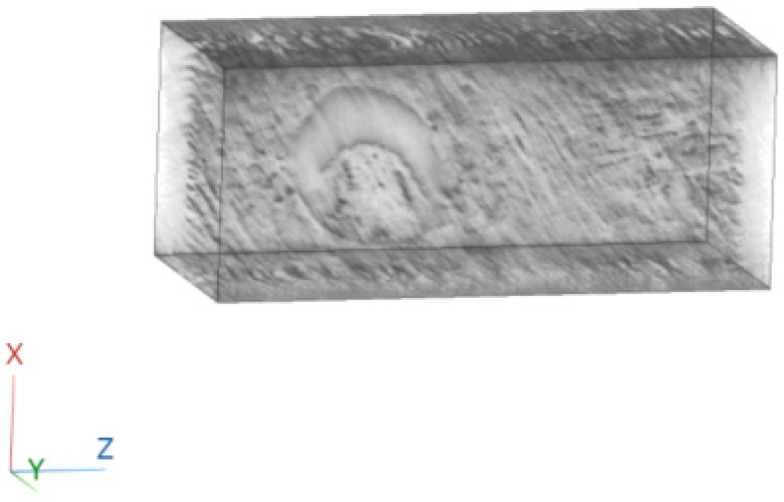
Three-dimensional reconstruction of a tissue section using ART.

Figure 17 shows the 3D reconstruction of the tissue section using the images obtained from the ART.

**Figure 18:**
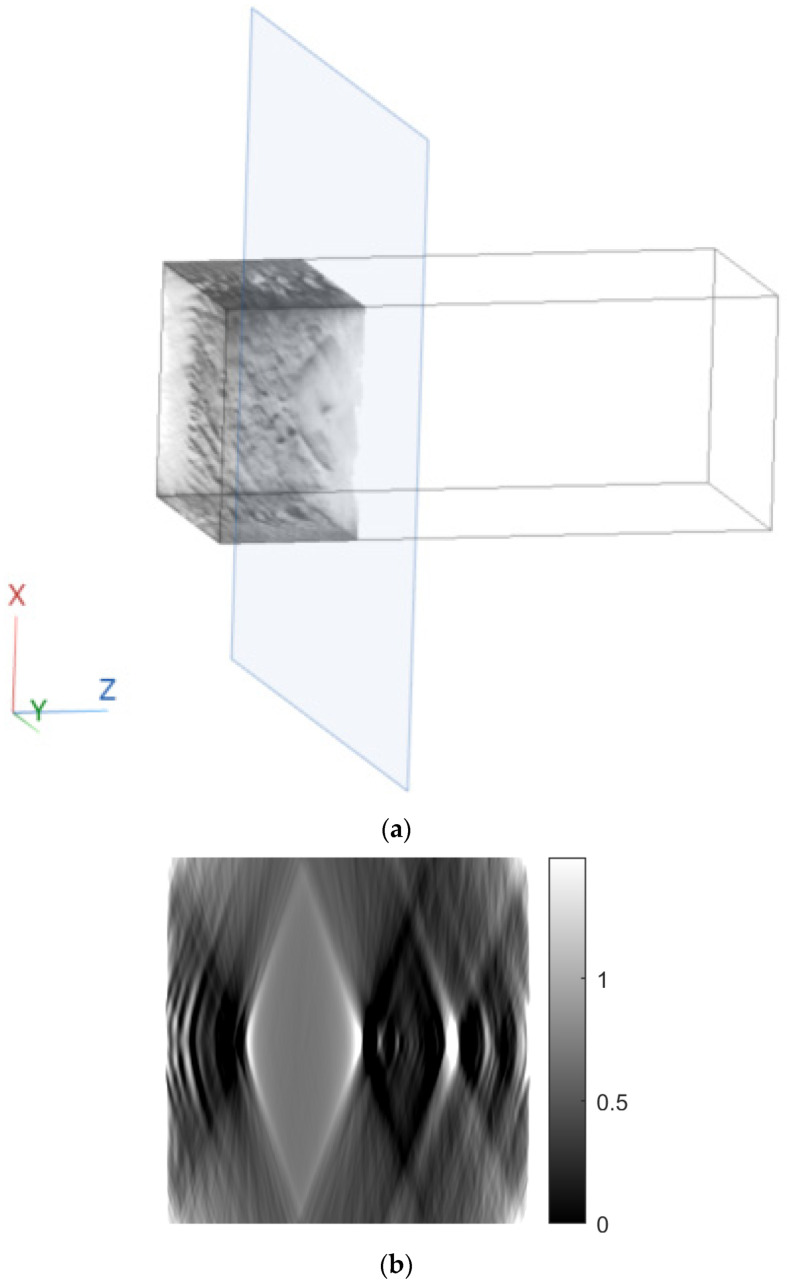
Example of an image in the axial plane using ART. (**a**) The plane in which the image was taken. (**b**) The image.

**Figure 19:**
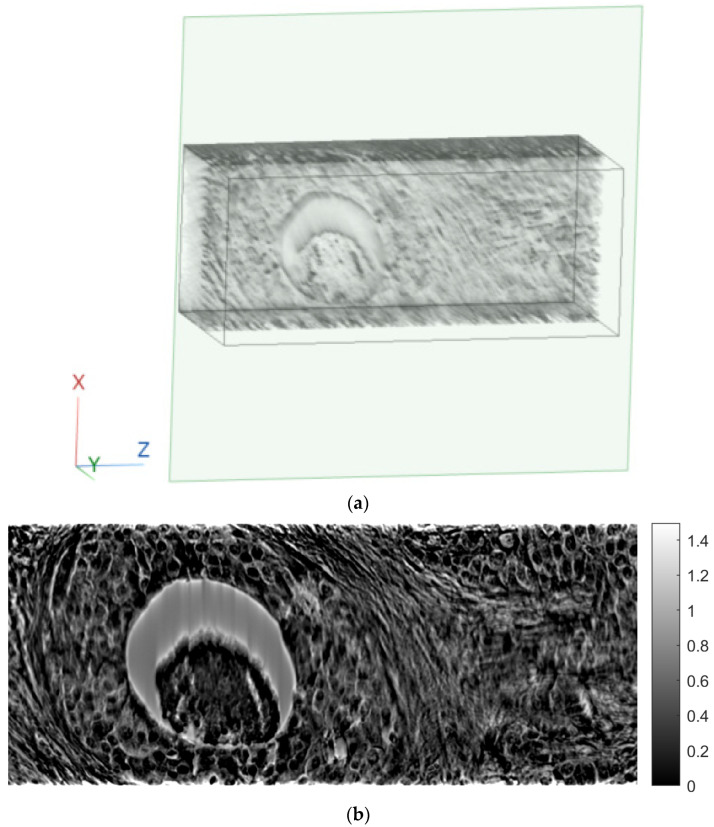
Example of an image in the coronal plane using ART. (**a**) The plane in which the image was taken. (**b**) The image.

**Figure 20:**
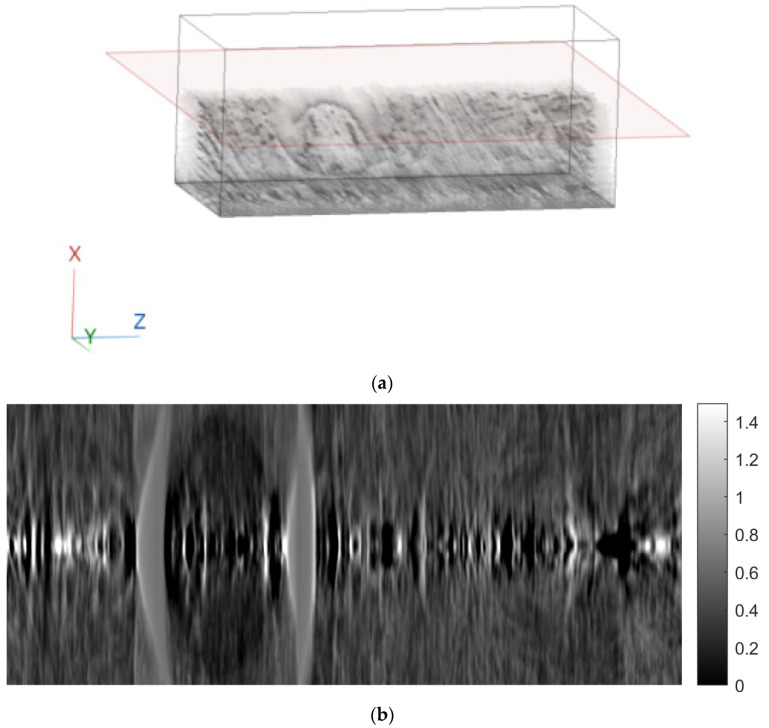
Example of an image in the sagittal plane using ART. (**a**) The plane in which the image was taken. (**b**) The image.

Figure 18, Figure 19 and Figure 20 provide indicative samples of images created using the ART in the axial, coronal, and sagittal planes, respectively. The 2D images depicted in Figure 18b, Figure 19b and Figure 20b were derived by slicing the volume at each corresponding plane indicated in Figure 18a, Figure 19a and Figure 20a, respectively.

**Figure 21:**
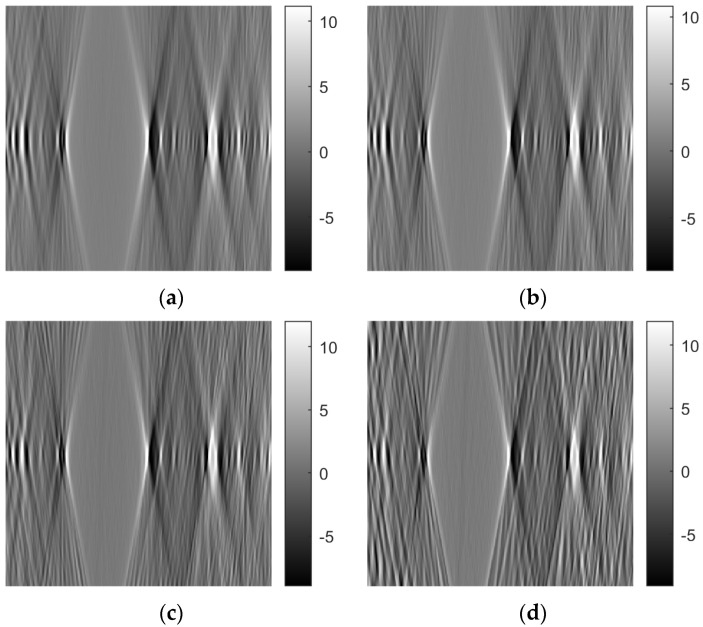
Comparison of reconstruction in the axial plane of the FBP for angle step: (**a**) 0.5°, **(b**) 1°, (**c**) 2°, (**d**) 4°.

**Figure 22:**
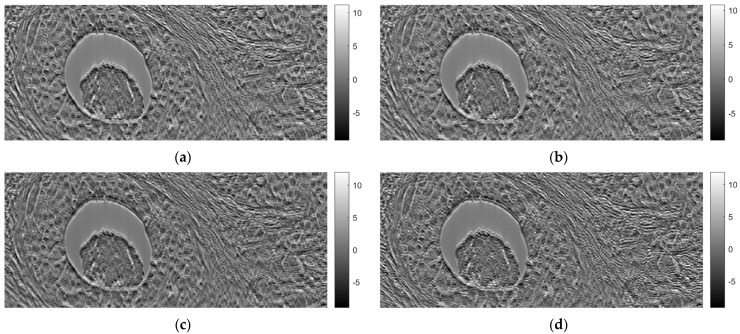
Comparison of reconstruction in the coronal plane of the FBP for angle step: (**a**) 0.5°, (**b**) 1°, (**c**) 2°, (**d**) 4°.

**Figure 23:**
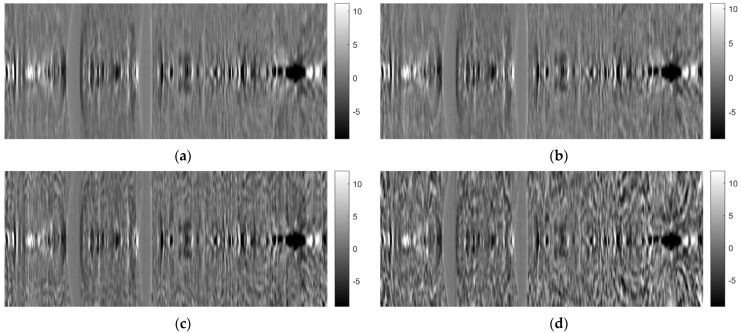
Comparison of reconstruction in the sagittal plane of the FBP for angle step: (**a**) 0.5°, (**b**) 1°, (**c**) 2°, (**d**) 4°.

Figure 21, Figure 22 and Figure 23 show images obtained from the FBP for the axial, coronal, and oblique planes, respectively, when different angle steps were used to obtain projections. In particular, results for angle steps of 0.5°, 1°, 2° and 4° are listed. As expected, as the angle step increases, the artifacts become more pronounced.

**Figure 24:**
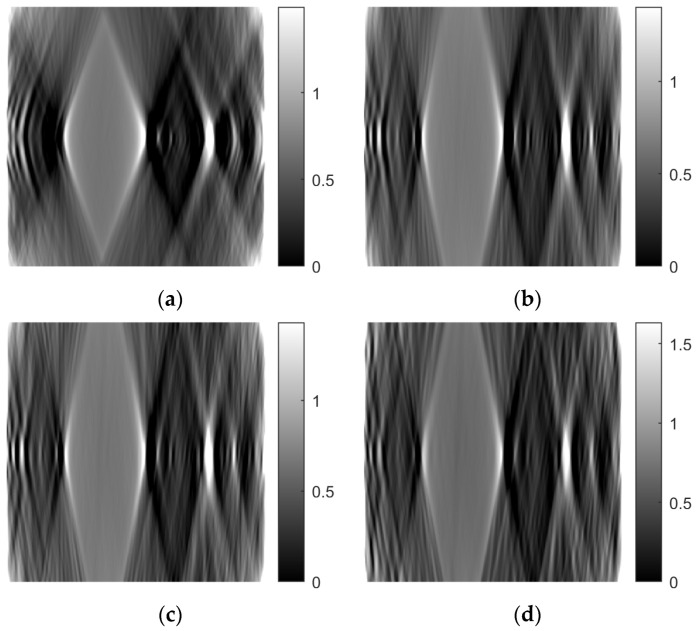
Comparison of reconstruction in the axial plane of the ART for angle step: (**a**) 0.5°, (**b**) 1°, (**c**) 2°, (**d**) 4°.

**Figure 25:**
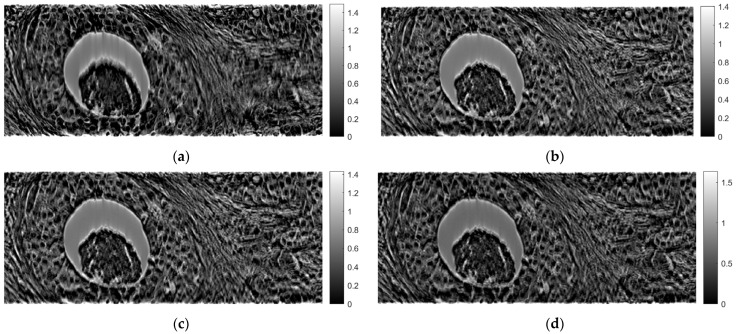
Comparison of reconstruction in the coronal plane of the ART for angle step: (**a**) 0.5°, (**b**) 1°, (**c**) 2°, (**d**) 4°.

**Figure 26:**
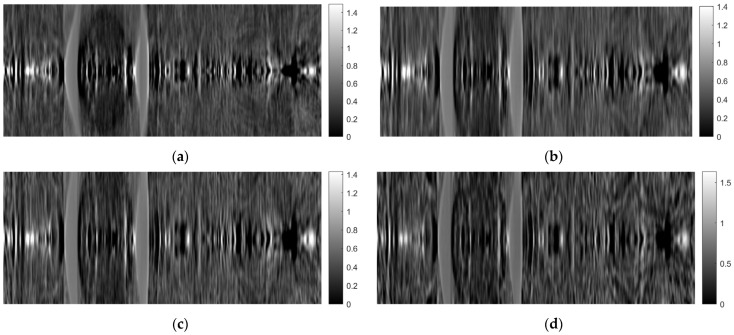
Comparison of reconstruction in the sagittal plane of the ART for angle step: (**a**) 0.5°, (**b**) 1°, (**c**) 2°, (**d**) 4°.

Figure 24, Figure 25 and Figure 26 illustrate images generated using the ART for the axial, coronal, and oblique planes, respectively, with varying angle step settings for acquiring projections. Specifically, results for angle steps of 0.5°, 1°, 2°, and 4° are presented. As anticipated, with increasing angle step size, artifacts become more noticeable, similar to what occurs in the FBP method.

**Figure 27:**
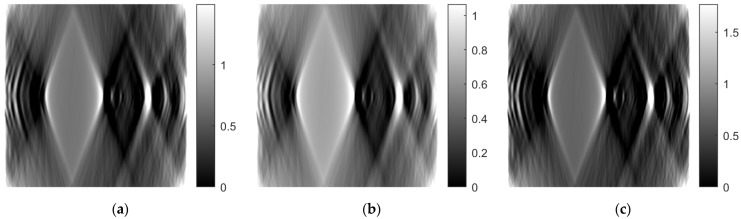
Comparison of reconstruction in the axial plane of the ART for (**a**) 10 iterations, (**b**) 5 iterations, and (**c**) 15 iterations.

**Figure 28:**
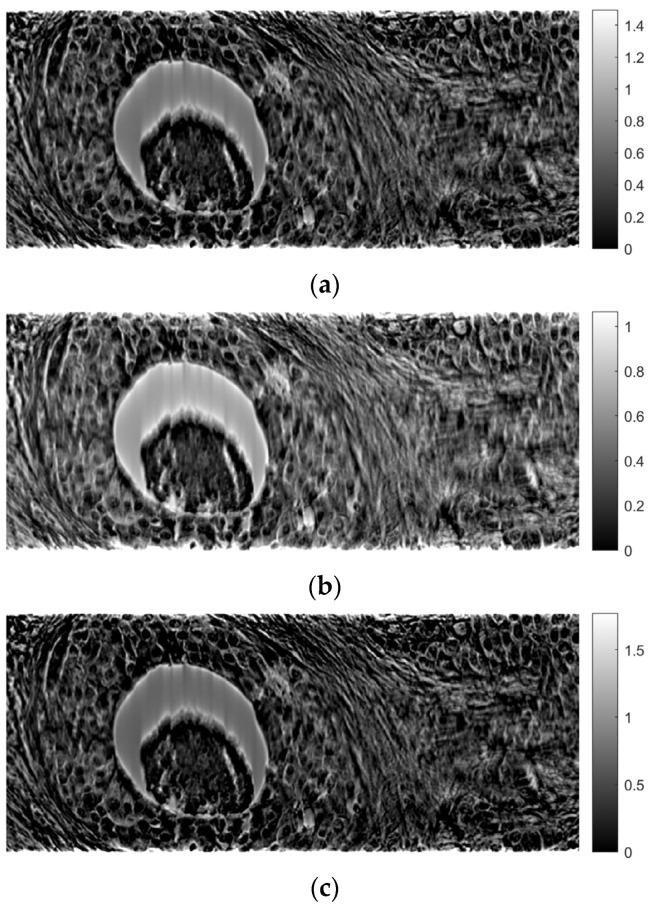
Comparison of reconstruction in the coronal plane of the ART for (**a**) 10 iterations, (**b**) 5 iterations, and (**c**) 15 iterations.

**Figure 29:**
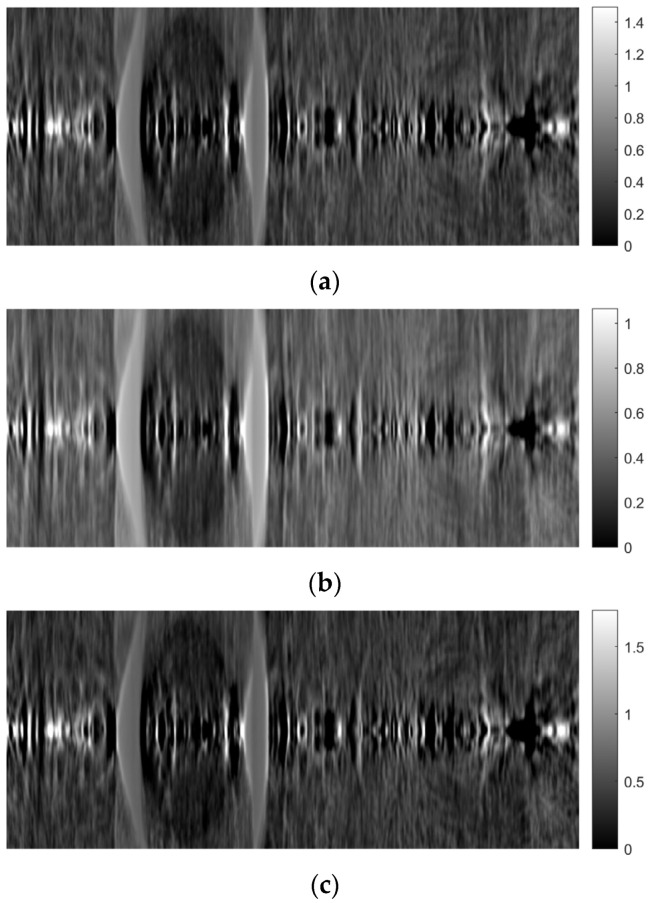
Comparison of reconstruction in the sagittal plane of the ART for (**a**) 10 iterations, (**b**) 5 iterations, and (**c**) 15 iterations.

As mentioned in the previous section, the ART was implemented using 10 iterations. Both 5 and 15 iterations were also tested. Figure 27, Figure 28 and Figure 29 show images obtained for the axial, coronal, and sagittal planes, respectively. No visual differentiation is observed. Therefore, reconstruction can be successfully performed, even with 5 iterations of ART.

## 4. Discussion

In this paper, a methodology for tomographic imaging of tissue samples using a bright field microscope was presented. The proposed methodology is based on the fact that each sample section has a finite thickness and, therefore, it is possible to create images at different levels within the section, revealing details that would probably not be seen otherwise. To achieve this goal, the basic principles of CT are used, in terms of reconstructing images from projections. Two classical reconstruction algorithms were tested: the filtered backprojection and the algebraic reconstruction techniques. The reconstruction was performed with a limited range of angles for obtaining projections. In particular, the range of angles was limited to between −10° and +10° due to physical constraints. The resulting images were of satisfactory quality, but, as expected, they exhibited some artifacts, which are particularly evident in the axial plane images (Figure 11b and Figure 18b). Comparing the two reconstruction methods, it appears that the ART produces images with slightly better contrast. In our scenario, which involves limited-angle or sparse data, the ART offers particular advantages by progressively refining image estimates through iterative data fitting. Although the ART requires higher computational resources than FBP, it can deliver enhanced image quality in this specific context.

Although the reconstructed images might not appear as sharp as their classical 2D projection counterparts, the clinical evaluation that we have performed in our previous study showed that the reconstructed images assisted the physician to discovered new information not apparent on 2D images [22]. Keeping in mind that the proof of concept regarding the clinical importance of our approach has been already highlighted in our previous study [22], in this study, we expand our efforts towards an approach that creates tomographic images without the need to physical slice the samples, as was the case in our previous work. Instead of physical slicing, in this study, we use optical slicing by designing and implementing a custom-made microscope stage, adaptable to commercial microscopes, designed to be compatible with the well-known computed tomography geometry. 

The concept of 3D optical tissue slicing has been explored in prior research efforts by various research groups [25,26,27,28,29,30,31,32,33,34,35]. Some of the most prominent technologies that have been proposed to tackle this issue are optical projection tomography (OPT) [36] and partially coherent optical diffraction tomography (PC-ODT) [37]. OPT requires a specialized instrument, constructed similarly to the X-ray CT system, for full sample rotation for the creation of 3D volume images. PC-ODT is a method that deconvolves and combines stacks of 2D images based on refractive index distribution measurements. 

However, previous studies [25,26,27,28,29,30,31,32,33,34,35] may exhibit certain limitations. Firstly, some of the methods proposed necessitate nearly complete sample rotation around the light source (or full rotation of the source around the sample), making them unsuitable for high magnifications that require the sample to be placed at a very close distance to the objectives (from a few mm to a few cm). Secondly, most of these methods cannot be integrated as a modular component onto standard brightfield (BF) microscopes, instead demanding the purchase of a dedicated, specialized instrument. Thirdly, while many of these methods have potential medical applications, only a few are realistically viable for clinical practice, despite being available on the market for over two decades.

In contrast, the proposed 3D tissue volume reconstruction methodology offers solutions to these limitations. Firstly, it is not reliant on the sample’s distance from the objectives and does not require full rotation of the sample. This allows for the production of 3D tissue volumes at any desired magnification, provided that the sample has a thickness close to the working distance of the selected objective. Secondly, it does not mandate the acquisition of a specialized instrument; it can be implemented with any regular brightfield microscope by replacing the microscope stage with another stage that additionally enables limited-angle rotation around the horizontal axis. Thirdly, this method is versatile and can be applied to a wide range of routine applications, not limited to specialized protocols.

However, the proposed method also has its limitations, the most important being that the reconstructed images sharpness does not reach the same quality as that of typical 2D images. This outcome was anticipated due to both the limited angular rotation and the small thickness of the sample (10 μm). Limited angular rotation is translated to limited number of projection data, necessitating the utilization of interpolation-based methods to complete the reconstruction task. The latter basically means that we can get a rough, blurry idea of the image content. One possible solution is to create new prototype designs of the glass slide on which the sample is mounted to enable a/a wider range of angular rotation, even when using higher magnifications where the glass slide and objective lens are in close proximity, and b/the accommodation of larger-sized samples, allowing for the generation of an increased number of projectional layers during the reconstruction process. Another limitation is that a special version of the proposed experimental platform is required to fit to the specific configuration of each different commercial microscope. This can be mitigated by creating a universal sample-holding microscopy stage that includes the capability for sample rotation. This approach would eliminate the need for attaching additional components to the microscope to enable 3D image reconstruction.

Since we are using a conventional halogen bulb, with wavelengths within the visual spectrum, an objective of ×10 with numerical aperture 0.25, and digital camera with actual pixel size 2.78 × 2.78 μm, the resolution of the experiment may be roughly considered within the range of 5–10 μm. Moreover, this resolution is further degraded in the reconstructed volumes due to interpolation and missing projectional data, since we are using a limited angular rotation of the sample. A more realistic estimation of the resolution of the full experiment, including the reconstruction process, could be obtained using a gold standard for comparison; for example, a specially designed phantom for which the ground truth would be known or a very thin tissue slice.

Another limitation of the study is the amount of out-of-focus light that is being recorded due the comparable size of depth of focus and tissue thickness, resulting in further resolution degradation in the image reconstruction calculations. In addition to the depth of focus limitation, comatic aberrations may further degrade image quality, increasing blurriness. If the application domain of the proposed set-up was to investigate small structures in the magnitude of few μm, then the proposed method would not produce reliable results due to the above-mentioned limitations. However, our application domain targets much larger structures in histopathology, such as nuclei, aggregations of nuclei, emboli, lumens, etc. To this end, we sought the assessment of an expert histopathologist evaluation (E.K.). The histopathologist acknowledged that the generated images lacked sufficient resolution and contrast. Nevertheless, despite these limitations, the histopathologist was still able to identify new information within the 3D images that was either challenging to observe or completely unidentifiable in the 2D images. From this perspective, the histopathologist found the reconstructed 3D images valuable as a complementary set of images to be presented alongside the conventional high-resolution and high-contrast 2D tissue images. 

One possible solution to the above limitations would be modification of the experimental platform to enable a/the controlled movement of the sample along the z-axis, which would produce more than one image for each projection, b/the removal of out-of-focus parts for each sub-image of the projection under investigation, and c/the stitching/fusing/combination of the clear parts of each sub-image in order to create the final image of the particular projection. Moreover, high-quality coma correction lenses may reduce the overall blurriness in the resulting reconstructed images.

Another possible solution to the above limitations would be to use tissue thicknesses larger than the depth of focus. In this way, we could eliminate, at least in theory, the depth of focus limitation. In the higher tissue thickness scenario, we would have to deal with an additional set of problems: a/staining is difficult to effectively penetrate tissues of higher thickness, which practically means that contrast will be reduced, b/thicker tissues are more difficult to accurately cut with the microtome, c/it would be difficult to fix higher thickness tissues on the conventional microscope glass slide. 

Despite all the above-mentioned challenges, the future goal for the evolution of this study is to adapt the approach towards a/elimination of the requirement of any staining in an analogous manner, such as in X-ray CT, b/investigation of tissues with thicknesses up to a few mm, until the penetration limit of visual light, and c/improvement of resolution down to the μm scale. 

## 5. Conclusions

In this work, a method for the tomographic reconstruction of tissue sample material was presented. The proposed method may be adjusted to any conventional bright field microscope by replacing the microscope’s stage with a new stage, enabling sample rotation around the horizontal axis. Using classical tomographic reconstruction algorithms at limited angles, it is possible to investigate the sample at any desired optical plane, revealing information that would be difficult to identify when focusing only on conventional 2D images. 

## Data Availability

Data are not available due to privacy and ethical restrictions.
